# The Aortic Flow Reversal Ratio: A Quantitative Adjunct to the Bicêtre Score in Vein of Galen Malformation

**DOI:** 10.3390/jcm15020748

**Published:** 2026-01-16

**Authors:** Menachem Rimler, Ranjit Philip, Lydia Tanner, Hannah Huth, Lucas Elijovich

**Affiliations:** 1 The Heart Institute, Le Bonheur Children’s Hospital, Memphis, TN 38103, USA; rphilip@uthsc.edu; 2 Division of Pediatric Cardiology, Department of Pediatrics, University of Tennessee Health Science Center, Memphis, TN 38103, USA; lhenry2@hs.uci.edu (L.T.);; 3 Department of Neurology, University of Tennessee Health Science Center, Memphis, TN 38103, USA; lelijovich@semmes-murphey.com; 4 Neurology, Semmes Murphey Clinic, Memphis, TN 38120, USA

**Keywords:** Vein of Galen Malformation (VGAM), aortic flow reversal ratio (AoFRr), Bicêtre score, systemic steal, echocardiography, endovascular embolization, neonate, Doppler, diastolic flow reversal, risk stratification

## Abstract

**Background/Objectives:** The Bicêtre score for Vein of Galen Aneurysmal Malformation (VGAM) relies on existing end-organ damage. We hypothesized that transthoracic echocardiography (TTE) could quantify significant systemic steal in clinically stable neonates (Bicêtre score ≥ 12). This study evaluates the Aortic Flow Reversal Ratio (AoFRr) as a tool to measure this steal and predict treatment outcomes. **Methods**: In a single-center retrospective study of patients with VGAM, the AoFRr (the ratio of the diastolic reversal velocity time integral to the systolic forward volume time integral) was calculated via TTE in the abdominal aorta at the level of the diaphragm before and after endovascular embolization. Over the study period, the cohort underwent a total of 30 endovascular interventions and 49 TTEs. Pre-intervention AoFRr was correlated with the Bicêtre score, and post-intervention changes were analyzed for association with the need for subsequent embolizations. **Results**: In a cohort of 12 patients with a median Bicêtre score of 18, 83.3% had pre-intervention aortic diastolic flow reversal. The median pre-intervention AoFRr was 0.81, indicating substantial systemic steal despite clinical stability. A post-intervention AoFRr reduction of ≥85% was significantly associated with a lower likelihood of requiring re-intervention (*p* = 0.0253). **Conclusions**: The AoFRr quantifies substantial hemodynamic steal in VGAM patients who appear clinically stable by the Bicêtre score. Its reduction following embolization predicts a more favorable clinical course. The AoFRr is a valuable, non-invasive adjunct for risk stratification and may help optimize the timing of endovascular intervention.

## 1. Introduction

**Figure 1:**
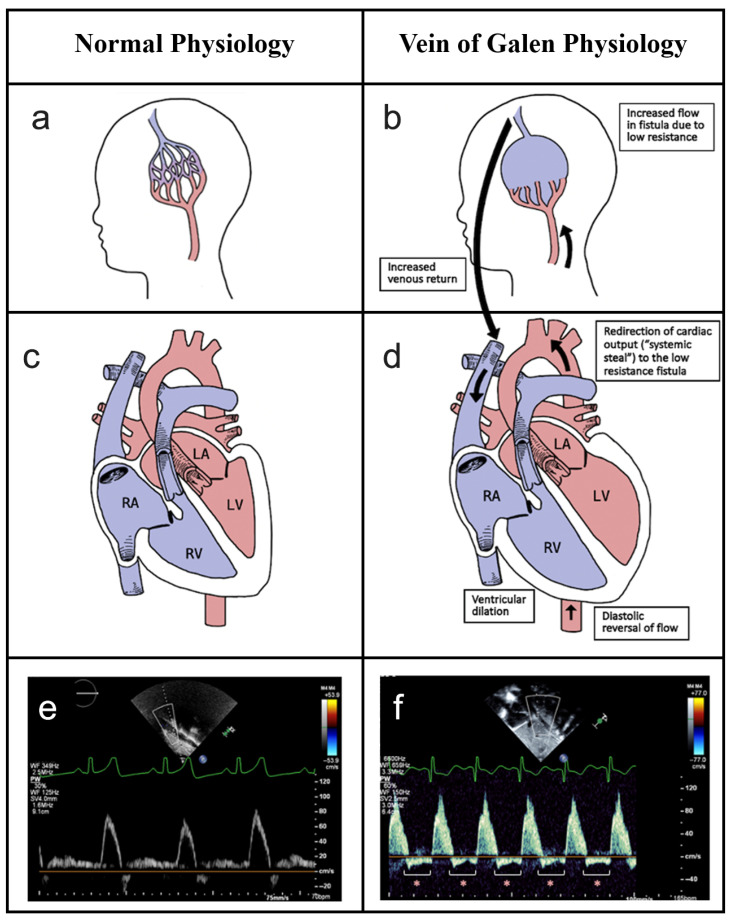
Pathophysiology of Vein of Galen Aneurysmal Malformation (**a**) Normal cerebral arterial and venous anatomy. (**b**) In VGAM, a low-resistance fistula shunts arterial blood directly into a persistent embryonic vein, leading to increased flow and venous return. (**c**) Normal cardiac physiology with balanced systemic circulation. (**d**) VGAM physiology demonstrates the consequences of the shunt, including redirection of cardiac output (“systemic steal”) toward the fistula, causing diastolic reversal of flow in the aorta and subsequent right-sided heart volume overload and ventricular dilation. (**e**) A normal Doppler flow pattern in the abdominal aorta shows only forward (anterograde) flow. (**f**) In VGAM, the Doppler pattern reveals substantial diastolic flow reversal (retrograde flow as indicated by red asterisks), indicative of aortic steal.

Vein of Galen Aneurysmal Malformation (VGAM) is a rare congenital intracranial arteriovenous malformation that shunts blood from arterial feeders to the persistent median prosencephalic vein of Markowski, a precursor to the vein of Galen [1,2,3,4,5]. This low-resistance shunt (Figure 1b) steals blood from the systemic circulation, a phenomenon known as systemic steal. This “steal” redirects cardiac output away from the body’s organs and toward the fistula, causing diastolic reversal of flow in the aorta (Figure 1d) and leading to significant hemodynamic disturbances [5,6]. Due to the redirection of cardiac output through the shunt, there is reduced perfusion of vital organs. The high velocity shunt bypasses normal gas exchange in the brain [7,8]. The consequent high venous return from the shunt causes right heart volume overload and high-output cardiac failure with decreased systemic perfusion despite a compensatory increase in cardiac output, which can be life-threatening in neonates [1,9,10,11] (Figure 1).

The hemodynamic effects can lead to respiratory distress, heart failure, and neurological abnormalities [12,13]. Without intervention, this condition can lead to multi-organ failure and death [1,2,14]. Endovascular embolization is the primary treatment modality aimed at reducing blood flow through the shunt [15,16]. However, the timing of intervention is critical, as both premature and delayed intervention can lead to complications [17,18,19].

The Bicêtre score is a clinical tool that evaluates multiple parameters (cardiac, respiratory, neurological, hepatic and renal function) and has traditionally been used to guide the neuro-interventionalist on optimal timing of the procedure [4,20]. It categorizes patients based on severity, with scores less than eight indicating a near-fatal prognosis and futility of intervention, scores between eight and 12 suggesting benefit from emergent endovascular embolization (EE), and scores greater than 12 indicating candidates for medical management until around 5 months of age. It is used as a proxy for grading end-organ damage. However, some centers have moved away from using the Bicêtre score as the sole determinant for therapy management [11,21,22,23].

Transthoracic echocardiograms (TTE) are routinely performed in neonates with VGAM to assess the hemodynamic changes resulting from the arteriovenous shunt, including pulmonary hypertension and high-output heart failure. It provides critical information on the severity of the condition and response to medical management [1,24,25,26].

**Figure 2:**
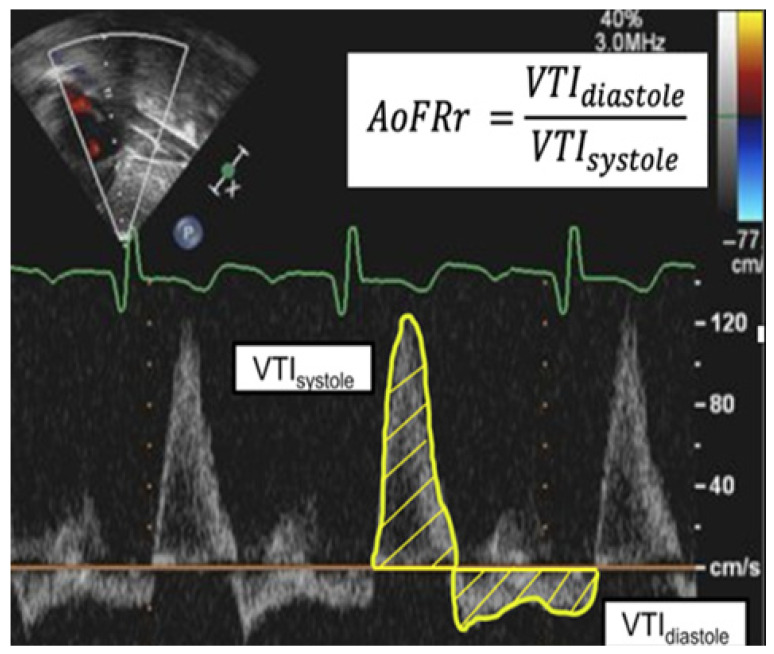
Echocardiographic Quantification of the Aortic Flow Reversal Ratio (AoFRr). A pulse-wave Doppler tracing from the abdominal aorta is used to calculate the AoFRr. The Velocity Time Integral (VTI), which is proportional to stroke volume, is measured by tracing the Doppler waveform. The systolic VTI (VTIsystole) represents the anterograde flow during systole (waveform above the baseline), while the diastolic reversal VTI (VTIdiastole) represents the retrograde flow stolen by the malformation during diastole (waveform below the baseline). The AoFRr is calculated as the ratio of these two values.

In these patients, systemic steal can be visualized on TTE as diastolic flow reversal in the aorta (Figure 1f), a stark contrast to the forward-only flow seen in normal physiology (Figure 1e) [27,28]. This reversal can be quantified using the Aortic Flow Reversal Ratio (AoFRr), which is calculated as the ratio of the diastolic reversal velocity time integral (VTI) to the systolic forward VTI (Figure 2). While the aortic flow reversal is a known surrogate for systemic steal in other conditions with shunts, such as a patent ductus arteriosus (PDA), its specific utility in VGAM remains poorly defined [29,30]. This represents a critical gap, as management decisions traditionally relied on clinical scores that reflect existing end-organ damage rather than the underlying hemodynamic stress. A reassuringly high Bicêtre score typically implies that the patient has an insignificant shunt that will not be problematic. However, it may also arise when a patient has a significant shunt but has not yet reached the point of maximal end-organ damage. This study focused on patients with a Bicêtre score > 12 at the time of embolization. The presence of flow reversal in this group suggests that the cohort likely includes patients fitting the latter scenario (significant shunt prior to end-organ damage), thereby demonstrating the inability of the Bicêtre score to reliably distinguish between hemodynamically insignificant and significant shunts. We hypothesized that TTE could quantify substantial systemic steal even in patients categorized as stable (Bicêtre score ≥ 12), thereby identifying a window for intervention, potentially before the onset of the end-organ damage detected by the clinical score.

## 2. Materials and Methods

We conducted a retrospective cohort study of patients treated at the University of Tennessee Health Science Center, Le Bonheur Children’s Hospital, Memphis, TN, over a 10-year period. We identified patients through a systematic review of institutional electronic medical records and radiology databases and verified the diagnosis of VGAM according to established diagnostic guidelines. To be included in this study, patients were required to meet the following criteria:

1. A confirmed diagnosis of VGAM based on imaging evidence.
2. A documented Bicêtre Neonatal Evaluation Score of greater than 12 at the time of initial assessment.
3. At least one EE procedure performed during the study period.
4. Availability of complete pre- and post-intervention TTE datasets.

We excluded patients with incomplete echocardiographic data or an unconfirmed diagnosis. The final selection process involved cross-referencing clinical notes with procedural records to confirm eligibility, ensuring that only infants with comprehensive and relevant data were included.

We organized data collection into two main categories, clinical and echocardiographic, each requiring specific methods to ensure accuracy and reliability.

Clinical Data: We evaluated the clinical condition before intervention using Bicêtre scores, a standardized system that measures multi-organ function (including cardiac, cerebral, respiratory, hepatic, and renal systems) in patients with VGAM. Clinicians extracted these scores from electronic medical records using a predefined procedure. We also recorded the patient’s age at intervention and the total number of EE procedures. The latter was used in our secondary analysis as a measure of treatment frequency and complexity.

Echocardiographic Data: A board-certified pediatric cardiologist reviewed TTE images obtained before and after each EE procedure for consistency. The primary focus was the AoFRr, a measurement used to assess the hemodynamic impact of VGAM. Doppler ultrasound of the abdominal aorta at the level of the diaphragm provides VTI measurements for both diastolic flow reversal (retrograde flow during diastole) and forward systolic flow (anterograde flow during systole). We calculated the diastolic reversal VTI by manually tracing the retrograde Doppler waveform and the systolic forward VTI by tracing the anterograde waveform. We then calculated the AoFRr as the ratio of the diastolic reversal VTI to the systolic forward VTI, expressed as a unitless value that correlates with the relative amount of shunted blood flow through the VGAM, as supported by prior studies of arteriovenous shunting [10,25,31,32,33] (Figure 2).

The primary outcome is to evaluate the pre-intervention flow reversal by TTE in patients with high Bicêtre scores. The secondary outcomes were (1) to correlate the Bicêtre score to the degree of reversal of flow, (2) to assess the hemodynamic impact of the intervention based on the change in the TTE-derived AoFRr by comparing the degree of change in AoFRr to the likelihood of undergoing additional EE.

We performed all statistical analyses using IBM SPSS Statistics v21.0 (RRID: SCR_016479) with statistical significance set at a *p*-value of less than 0.05. We used descriptive statistics to characterize the study cohort. Continuous variables, such as the pre-intervention AoFRr, are presented as medians and interquartile ranges (IQR). Categorical data, including the presence or absence of flow reversal, are reported as percentages. We determined the statistical significance of the percentage decrease in AoFRr following the initial endovascular procedure using a Wilcoxon signed-rank test for paired samples. To evaluate the secondary outcomes, we conducted the following analyses:

1. A simple linear regression to assess the relationship between the pre-intervention AoFRr and the Bicêtre score, with the coefficient of determination (R^2^) reported to quantify the correlation.
2. An assessment of the association between the reduction in flow and the number of subsequent interventions using two methods. First, we calculated Spearman’s rank correlation to measure the relationship between the ranked absolute flow reduction (AFR) and the total number of re-interventions. Second, we dichotomized the percentage drop in AoFRr using an 85% reduction as a cutoff and used a Chi-square test of independence to determine its association with the likelihood of requiring any re-intervention.

## 3. Results

### 3.1. Patient Demographics and Clinical Characteristics

A total of 12 patients met the inclusion criteria for this study. During the study period, no patients meeting the inclusion criteria were excluded. The cohort was predominantly male (*n* = 7, 58.3%). The median age at the time of the initial endovascular intervention was 3.7 months (IQR 0.3–39.5 months), with a median weight of 3.4 kg (IQR 3.0–6.2 kg) and a median height of 51.5 cm (IQR 50.0–54.0 cm). Over the study period, the cohort underwent a total of 30 endovascular interventions and 49 TTEs. The median number of interventions per patient was two (IQR 1–3), and the median number of echocardiograms per patient was five (IQR 3–8) (Table 1).

All interventions were performed via a transfemoral arterial route using liquid embolic embolization of the VGAM fistulous connections with n-butyl cyanoacrylate. As per the study’s inclusion criteria, all patients had a pre-intervention Bicêtre Neonatal Evaluation Score greater than 12. The median score for the cohort was 18 (IQR 15.5–20). The median scores for the clinical subcategories were as follows: Cardiac 5 (IQR 2.5–5), Cerebral 5 (IQR 5–5), Respiratory 5 (IQR 2.5–5), Hepatic 3 (IQR 3–3), and Renal 3 (IQR 3–3) (Table 2).

### 3.2. Baseline Echocardiogram Findings and Correlation with Bicêtre Scores

Prior to the initial endovascular intervention, diastolic flow reversal in the abdominal aorta was a common finding, present in 83.3% of the patients. The median pre-intervention AoFRr for the cohort was 0.81 (IQR 0.49–1.05), indicating a degree of systemic steal even in patients with high Bicêtre scores. A simple linear regression analysis demonstrated a moderate negative correlation between the pre-intervention AoFRr and the total Bicêtre score, yielding an R-squared value of 0.4546 (Table 3).

### 3.3. Hemodynamic Impact of Endovascular Intervention

The initial EE procedure had a significant hemodynamic impact, resulting in a mean percentage decrease in the AoFRr of 52.80% (*p* = 0.0232). The degree of flow reduction after the first intervention was associated with the need for subsequent procedures. Spearman’s rank correlation showed a moderate negative relationship between the absolute flow reduction (AFR) and the number of re-interventions (ρ ≈ −0.55, where ρ represents Spearman’s rank correlation coefficient). Furthermore, when the percentage drop in AoFRr was dichotomized at a cutoff of 85%, a Chi-square test revealed that patients achieving this level of reduction were significantly less likely to require any re-intervention (χ^2^ = 5, *p* = 0.0253) (Table 3).

## 4. Discussion

Infants with VGAM present significant challenges for clinicians in prognostication and management. The Bicêtre score is a valuable clinical tool used to assess the severity of heart failure and multi-organ dysfunction. This study focuses on the adjunct use of TTE as a non-invasive metric to evaluate the hemodynamic significance of VGAM in providing an insight into not only the timing of intervention but also the hemodynamic effect of the intervention. TTE is an essential, non-invasive tool in the initial diagnosis and ongoing management of neonates with VGAM. Its primary role has been to evaluate the profound hemodynamic consequences of the high-flow intracranial shunt, most notably high-output cardiac failure [25]. Previous studies have consistently identified hallmark structural findings on TTE, including dilation of the right atrium and ventricle, flattening of the interventricular septum, and a dilated superior vena cava due to the massive venous return from the malformation [34,35,36] (Figure 1b,d). Beyond structural assessment, Doppler ultrasonography is critical for identifying prognostic markers [10,25,32,37]. The phenomenon of diastolic flow reversal in the descending aorta, or “aortic steal,” is a well-documented qualitative marker of a significant shunt [29,30,31] (Figure 1f). To better quantify the degree of systemic steal, we utilized the VTI (Figure 2). Physically, the VTI represents the linear distance that a column of blood travels during a single cardiac cycle. It is mathematically defined as the integral of the velocity curve over the duration of the flow period:

$$VTI=\;\int_{0}^{T}v(t)\;dt$$

In a pulsatile system like the aorta, the stroke volume (SV) is the product of the vessel’s cross-sectional area (CSA) and the VTI (SV=CSA×VTI). Therefore, VTI indicates the volume of blood flowing in a given direction within a particular vessel during a beat [38,39,40]. Schwarz et al. (2024) proposed a “percentage of steal” using the time-averaged maximum velocity (TAMAX) to represent retrograde flow as a proportion of total flow (Retrograde TAMAX)/(Antegrade TAMAX + Retrograde TAMAX) [32]. TAMAX is mathematically defined as the velocity time integral divided by the duration of flow ($T_{flow}$):

$$TAMAX=\frac{\int_{0}^{T}v\left(t\right)\;dt}{T_{flow}}=\frac{VTI}{T_{flow}}$$

While TAMAX is an excellent metric for characterizing flow velocities and vascular resistance, we utilized the VTI directly to calculate the AoFRr because it provides a direct volumetric comparison for pulsatile flow. Assuming the aortic diameter remains constant between systole and diastole, the CSA terms cancel out, leaving the ratio of Retrograde to Antegrade VTI as a dimensionless, direct proxy for the volumetric ratio:

$$AoFRr\;=\;\frac{VTI_{retro}}{VTI_{ante}}$$

Furthermore, because VTI tracing is a fundamental skill in sonography and a common function on modern ultrasound systems, this metric can be readily adopted and consistently measured across different institutions without requiring specialized software or complex calculations.

We deliberately selected the antegrade VTI as the denominator (rather than the sum of antegrade and retrograde flows) because the sum of forward and backward flow in the abdominal aorta does not equate to a standard physiological parameter. In contrast, the antegrade VTI represents the volume of blood effectively delivered to the lower body. Therefore, our ratio isolates the magnitude of the “stolen” volume relative to the “delivered” volume. This VTI-based approach is similar to the method described by Doctor et al. (2024), who used an Antegrade/Retrograde ratio [25]. However, we chose to utilize the inverse (Retrograde/Antegrade) for two critical reasons. First, the Doctor et al. (2024) [25] ratio becomes mathematically undefined in normal physiology, where retrograde flow is zero (division by zero). Our metric avoids this instability, yielding a value of 0 in normal patients. Second, our ratio may be more clinically intuitive, as a higher number directly corresponds to greater pathology (more steal).

There is growing evidence linking the degree of flow reversal with mortality. Using their metric, Doctor et al. (2024) found that an antegrade-to-retrograde VTI ratio (Antegrade VTI/Retrograde VTI) less than 1.5 was predictive of mortality [25]. Recently, Schwarz et al. (2025) also demonstrated the prognostic value of aortic steal using a percentage-based metric derived from TAMAX, showing correlations between degree of steal and neurodevelopmental outcomes at age two years [41].

Unfortunately, direct comparisons between our dataset and that of Doctor et al. (2024) [25] and Schwarz et al. (2025) [41] are complicated by the different calculation techniques. While a direct mathematical conversion between our VTI-based AoFRr and the TAMAX-based ‘percentage of steal’ used by Schwarz et al. (2025) [41] is not possible due to the different underlying measurements, both ratios serve the same fundamental purpose: to quantify the proportion of retrograde flow relative to antegrade flow as a surrogate for the severity of the systemic steal. The metric of Doctor et al. (2024) [25] on the other hand is simple to convert to our AoFRr as our formula is simply the inverse of theirs. Therefore, the mortality-predictive threshold of <1.5 reported by Doctor et al. (2024) [25] (an antegrade-to-retrograde ratio) is mathematically equivalent to an AoFRr > 0.67 in our metric. Note that inverting their threshold value (1/1.5 ≈ 0.67) also requires the inequality sign to flip (from < to >).

Another difficulty when comparing our results to those of the previously mentioned studies is that our flow measurements were obtained in the descending abdominal aorta at the level of the diaphragm, while Schwarz et al. (2025) [41] measured flow reversal in the aortic isthmus and Doctor et al. (2024) [25] measured the flow reversal in the descending thoracic aorta. Due to the potential confounding of PDA shunting, pre-ductal and post-ductal measurements might not be directly comparable. Both measurement points have distinct physiological implications. Pre-ductal measurements may better reflect the steal effect on the brain parenchyma because the steal cannot be compensated for by right-to-left shunting across a PDA. Conversely, post-ductal measurements, as used in this study, may better reflect the effective perfusion pressure delivered to the lower body. While an open PDA with right-to-left shunting can mitigate the degree of measured reversal in the abdominal aorta, this location remains a valid proxy for systemic hypoperfusion distal to the heart. Thus, the optimal measurement point depends on the specific endpoint of the study.

The principle of using aortic diastolic flow reversal as a surrogate for systemic steal is not unique to VGAM and is a well-established concept in the assessment of other hemodynamically significant shunts, such as a PDA [30,33,42]. In the presence of a moderate to large PDA with low pulmonary vascular resistance, one of the expected findings is holo-diastolic flow reversal at the descending aorta [29,31]. Reinforcing its value in assessment of hemodynamic significance, a study by Broadhouse et al. (2013) investigating various echo markers in comparison to cardiac MRI, found that diastolic flow reversal in the descending aorta had the best correlation with a hemodynamically significant PDA when compared to other parameters [42].

The primary finding of this investigation is the profound disconnect between clinical appearance and underlying hemodynamic reality in infants with VGAM. We found that marked systemic steal, as quantified by the AoFRr, is highly prevalent even in a cohort considered clinically stable with a Bicêtre score of 12 or greater. While the Bicêtre score remains an invaluable tool for assessing overt, end-stage multi-organ dysfunction, our results suggest its shortcomings as an early diagnostic tool for hemodynamic significance. An overwhelming majority of these “stable” patients (83.3%) exhibited flow reversal with a median pre-intervention AoFRr of 0.81, indicating that 81% of the blood pumped towards the lower body was stolen back into the low-resistance shunt. This AoFRr of 0.81 would be equivalent to 1.23 using the Doctor et al. (2024) [25] metric. Per their findings, this degree of flow reversal would be associated with a higher mortality risk.

This highlights a critical insight: a reassuring Bicêtre score can mask a state of significant, ongoing hemodynamic stress. The AoFRr provides a direct, non-invasive measure of this systemic steal, offering a quantitative window into the underlying pathophysiology. It allows clinicians to see the fraction of cardiac output being diverted from the body long before this chronic burden translates into the overt clinical end-organ damage required to lower the Bicêtre score. This finding directly addresses a key gap in risk stratification, demonstrating that the AoFRr can unmask high-risk hemodynamic compromise in a subclinical phase. It therefore has the potential to serve as a more sensitive, early indicator to guide the timing of intervention, suggesting that endovascular embolization could be beneficial in mitigating this silent burden even in patients who appear clinically well. This indicator would need to be weighed against the known risks of early embolization.

Perhaps the most clinically actionable finding of this study is the association between the post-procedural reduction in AoFRr and the need for future interventions. The initial endovascular embolization resulted in a statistically significant mean decrease in the AoFRr of 52.80% (*p* = 0.0232). This intervention effectively reduced the cohort’s median AoFRr from a high-risk value of 0.81 to approximately 0.38—a level well below the critical threshold of 0.67 derived from the Doctor et al. (2024) data [25]. This is not merely a statistical change; it represents a clear and quantifiable hemodynamic improvement, reflecting a dramatic reduction in the steal phenomenon and a restoration of more effective systemic perfusion. This immediate, measurable impact on aortic flow dynamics is what gives the metric its profound clinical utility. The magnitude of this improvement has a dual utility:

1. Determining Procedural Success: For the interventionalist, the change in AoFRr offers immediate, objective feedback on the hemodynamic success of an embolization. It redefines procedural success, shifting the focus from simple anatomical occlusion to quantifiable physiological improvement. Achieving a near-complete reduction in the AoFRr (e.g., ≥85% as identified in our analysis) could serve as a novel therapeutic endpoint for the initial procedure. However, this target must be pursued with caution. Rapidly eliminating a high-flow shunt carries the risk of Normal Perfusion Pressure Breakthrough, where the sudden restoration of perfusion pressure can increase the risk of hemorrhagic complications. Thus, while a reduction of ≥85% predicts a favorable course regarding re-intervention, the interventionalist must balance this efficacy against the safety profile of staged embolization.
2. Prognostication and Family Counseling: For both the clinical team and the patient’s family, the magnitude of the post-procedural drop in AoFRr is a powerful prognostic tool. It allows for more informed counseling regarding the expected clinical course. A large reduction can provide reassurance and potentially suggest a lower likelihood of requiring further interventions, whereas a minimal reduction can help set expectations that, despite a technically successful procedure, further EE will likely be necessary.

This study’s findings are constrained by several factors. Its retrospective, single-center design and a small cohort limit statistical power and generalizability. Additionally, the measurement of flow reversal in the abdominal aorta can be influenced by other shunts, specifically a PDA [29,30,31]. However, the effect of the PDA depends on the direction of ductal shunting. A left-to-right shunt contributes to diastolic runoff, potentially leading to an overestimation of the steal attributable solely to the VGAM. Conversely, a right-to-left shunt (which is common in VGAM due to pulmonary hypertension) provides antegrade flow to the descending aorta, potentially masking the severity of the intracranial shunt (and leading to underestimation). Theoretically, measuring proximal to the PDA would mitigate this effect and provide a more accurate measure of VGAM shunt. However, regardless of the ductal flow direction, the post-ductal measurement more directly reflects the net effective flow delivered to the lower body.

Furthermore, a significant selection bias was introduced by including only patients with a Bicêtre score greater than 12. This focus was necessary as a review of institutional records revealed an insufficient number of patients with a score of 12 or less to perform a meaningful comparative analysis. Consequently, our conclusions regarding the AoFRr cannot be extrapolated to the entire VGAM population, particularly the most critically ill infants, and require validation in larger, multi-center prospective studies. Additionally, our secondary outcomes focused on a procedural endpoint (need for re-intervention) rather than long-term clinical data. Although other studies have linked different aortic flow reversal metrics to clinical outcomes, we cannot establish a direct association between AoFRr and neurodevelopmental outcomes due to methodological differences in our data collection.

While this study provides valuable preliminary evidence for the utility of the AoFRr in a select population of infants with VGAM, several avenues for future research are necessary to validate and expand upon these findings.

First, a large-scale, multi-center prospective study is the critical next step. This future research should aim to enroll patients across the entire clinical spectrum of VGAM, including those presenting with severe heart failure and low Bicêtre scores. By analyzing the AoFRr in a more heterogeneous population, a more robust risk-stratification model could be developed, potentially identifying different hemodynamic thresholds for intervention based on the initial clinical severity and VGAM subtype (choroidal vs. mural). This future trial could consider measuring the flow reversal at the aortic isthmus to minimize the impact of steal from a PDA.

Furthermore, if AoFRr is to be adopted clinically, its correlation with long-term neurodevelopmental outcomes must be established rather than inferred from similar but distinct TAMAX-based metrics. Prospective longitudinal studies following patients from infancy through childhood are essential to determine whether early correction of hemodynamic steal translates into improved long-term outcomes.

Ultimately, the findings from this and subsequent observational studies could provide the foundation for a prospective interventional trial. While the Bicêtre score has historically guided management, its well-known limitations have led some centers to move away from its exclusive use. A future trial would aim to determine if a treatment algorithm integrating the AoFRr or other metrics of aortic flow reversal could lead to improved outcomes. This approach could help refine the management paradigm by identifying a window for proactive intervention based on quantifiable hemodynamic data, potentially before the clinical decline historically required to trigger treatment. This line of inquiry could lead to more personalized and timely interventions, with the goal of improving the overall prognosis for this vulnerable patient population.

## 5. Conclusions

The AoFRr is a non-invasive metric that unmasks severe hemodynamic derangement in infants who otherwise appear clinically stable. As an adjunct to other indicators (such as the traditional Bicêtre score), it supports proactive intervention based on hemodynamic severity rather than waiting for end-organ failure. Furthermore, a post-procedural AoFRr reduction of ≥85% serves as a quantifiable target for success, predicting a significantly lower need for re-intervention. Integrating this metric into routine assessment shifts the paradigm from reactive treatment to hemodynamically guided management.

## Figures and Tables

**Table 1:** Patient Demographics and Procedural Characteristics of the Study Cohort.

Demographics	
Age at Initial Intervention (Months)	3.7 (0.3–39.5)
Weight at Initial Intervention (kg)	3.4 (3.0–6.2)
Height at Initial Intervention (cm)	51.5 (50.0–54.0)
Number of Interventions: total, median (IQR)	30, 2 (1.8–3.0)
Number of Echocardiograms: total, median (IQR)	49, 5 (3–8)
Male: *n* (%)	7 (58.3%)
Data above are Median (Interquartile Range) unless otherwise specified	

**Table 2:** Individual Patient Demographics, Bicêtre Score Components, and Aortic Flow Reversal Ratios.

ID	Sex (F/M)	Age	# of Interventions	Bicêtre Score Components					Total Bicêtre Score	AoFRr	
				Cardiac	Cerebral	Respiratory	Hepatic	Renal		Pre-Intervention	Post-Intervention
1	M	64 m	1	5	5	5	3	3	21	0.82	0.65
2	F	28 m	5	5	2	5	3	3	18	0.92	0.00
3	M	1 d	2	1	5	1	3	3	13	1.04	0.66
4	F	4 m	2	5	5	5	2	3	20	0.48	0.51
5	F	19 d	1	1	5	5	3	3	17	0.49	0.00
6	M	11 d	3	3	5	5	3	2	18	0.81	0.51
7	M	2 d	3	3	5	1	2	3	14	1.20	0.60
8	M	1 d	5	1	5	1	3	3	13	0.93	0.56
9	M	*	2	5	5	5	3	3	21	0.00	0.00
10	M	*	3	5	0	5	3	3	16	0.86	0.00
11	F	32 m	2	5	5	3	3	3	19	0.00	0.00
12	F	4 m	1	5	5	4	3	3	20	0.14	0.32

AoFRr = Aortic Flow Reversal Ratio. Age reported in days (d) or months (m) at time of initial intervention. * Age withheld to protect patient privacy.

**Table 3:** Summary of Aortic Flow Reversal Ratio (AoFRr) Findings and Outcomes.

Results	
Percentage of patients with reversal of flow	83.3%
Pre-intervention AoFRr: Median (IQR)	0.81 (0.49–1.05)
Correlation of Pre-intervention AoFRr with Bicêtre Score: R-Squared	0.4546
Percentage decrease in degree of AoFRr after intervention: Mean (*p*-value)	52.80% (0.0232 *)
Absolute Flow Reduction (AFR) vs. Number of Re-interventions: (ρ)	≈−0.55

* indicates a statistically significant difference with a *p* value < 0.05.

## Data Availability

The datasets generated and analyzed during the current study are not publicly available due to patient privacy and confidentiality but are available from the corresponding author on reasonable request.

## Abbreviations

The following abbreviations are used in this manuscript:

AFR	Absolute Flow Reduction
AoFRr	Aortic Flow Reversal Ratio
EE	Endovascular Embolization
PDA	Patent Ductus Arteriosus
TAMAX	Time-Averaged Maximum Velocity
TTE	Transthoracic Echocardiography
VGAM	Vein of Galen Aneurysmal Malformation
VTI	Velocity Time Integral
